# Stoichiometric homeostasis of N:P ratio drives species-specific symbiotic N fixation inhibition under N addition

**DOI:** 10.3389/fpls.2023.1076894

**Published:** 2023-04-26

**Authors:** Qiang Li, Joshua Philp, Matthew D. Denton, Yingxin Huang, Jian Wei, Huijuan Sun, Yang Li, Qian Zhao

**Affiliations:** ^1^ Northeast Institute of Geography and Agroecology, Chinese Academy of Sciences, Changchun, China; ^2^ Jilin Provincial Key Laboratory of Grassland Farming, Science and Technology Department of Jilin Province, Changchun, China; ^3^ School of Agriculture, Food and Wine, University of Adelaide, Glen Osmond, SA, Australia; ^4^ College of Life Sciences, Changchun Normal University, Changchun, China; ^5^ College of Forestry and Grassland Science, Jilin Agricultural University, Changchun, China

**Keywords:** stoichiometry, N:P ratio, symbiotic N fixation, rhizosphere, phosphorus mobilization, herbaceous legume

## Abstract

**Introduction:**

Symbiotic N fixation inhibition induced by N supply to legumes is potentially regulated by the relative N and P availability in soil. However, the specific responses of different legume species to changes in N:P availability remain unclear, and must be better understood to optimize symbiotic N fixation inputs under N enrichment. This study investigated mechanisms by which soil N and P supply influence the symbiotic N fixation of eight legume species, to quantify the inter-specific differences, and to demonstrate how these differences can be determined by the stoichiometric homeostasis in N:P ratios (H_N:P_).

**Methods:**

Eight herbaceous legume species were grown separately in outdoor pots and treated with either no fertilizer (control), N fertilizer (14 g N m^-2^), P fertilizer (3.5 g P m^-2^) or both N and P fertilizer. Plant nutrients, stoichiometric characteristics, root biomass, non-structural carbohydrates (NSC), rhizosphere chemistry, P mobilization, root nodulation and symbiotic N fixation were measured.

**Results:**

N addition enhanced rhizosphere P mobilization but drove a loss of root biomass and root NSC *via* exudation of P mobilization compound (organic acid), especially so in treatments without P addition. N addition also induced a 2-14% or 14-36% decline in symbiotic N fixation per plant biomass by legumes in treatments with or without P addition, as a result of decreasing root biomass and root NSC. The changes in symbiotic N fixation were positively correlated with stoichiometric homeostasis of N:P ratios in intact plants without root nodules, regardless of P additions.

**Discussion:**

This study indicates that N addition can induce relative P limitations for growth, which can stimulate rhizosphere P mobilization at the expense of root biomass and carbohydrate concentrations, reducing symbiotic N fixation in legumes. Legume species that had less changes in plant N:P ratio, such as *Lespedeza daurica and Medicago varia* maintained symbiotic N fixation to a greater extent under N addition.

## 1 Introduction

Symbiotic nitrogen (N) fixation by leguminous plants contributes significant N to the global biosphere, improving the productivity and sustainability of global agriculture (Herridge et al., 2008; Vitousek et al., 2013; Farrer et al., 2016; Li Q. et al., 2016). However, agricultural activities considerably increase the availability of soil N in many ecosystems worldwide (Galloway, 1998), inhibiting symbiotic N fixation by legumes (West et al., 2005; Nyfeler et al., 2011; Zheng et al., 2019). One mechanism for this inhibition is the legume’s preference for lower-cost strategies of soil mineral N uptake, in preference to the metabolically expensive option of nodule development and N fixation (West et al., 2005). Furthermore, N enrichment potentially increases the nitrate concentration in soil, which may directly inhibit root nodulation, nodule growth, and nitrogen fixation activity by reducing the production of flavonoids involved in Nod factor synthesis (Nanjareddy et al., 2014; Nishida and Suzaki, 2018), down-regulating expression of key genes controlling the development of nodule vasculature, and accelerating nodule senescence or disintegration (Matamoros et al., 1999). These mechanisms indicate that N fixation inhibition due to increased soil N availability could be common phenomena. However, some researchers have identified that N enrichment does not always downregulate symbiotic N fixation, even under high N supply, implying that additional factors may modify the effect of soil N enrichment on symbiotic N fixation (Zheng et al., 2019).

Soil phosphorus (P) status may be a key abiotic factor that regulates the effect of soil N enrichment on symbiotic N fixation, given the widespread co-limitation of terrestrial plant functions by N and P availability (Craine et al., 2008; Harpole et al., 2011). Legumes particularly require P for growth, root development, and formation of nodules with symbionts (Batterman et al., 2013; Sulieman et al., 2013), and the symbionts also demand P-rich ATP molecules to facilitate the high energy cost of the nitrogenase reaction (Augusto et al., 2013; Divito and Sadras, 2014). Increasing P availability in soil can improve symbiotic N fixation across multiple sites (Augusto et al., 2013).

The coupling of N and P requirements during plant growth and physiological metabolism can cause changes in either N or P availability to alter plant dependence on the other nutrient (Reich et al., 2009; Peñuelas et al., 2013). For example, N enrichment can increase plant P demands, accelerating the depletion of soil P (Gusewell, 2004; Png et al., 2017), and exacerbating plant P deficiency. Under the combination of low P availability and N enrichment, the imbalance between N and P supply are likely to elicit additional mechanisms that reduce symbiotic N fixation. N enrichment typically depresses root nodulation of legumes under low P availability, but enhances root nodulation under higher P availability (Gates and Wilson, 1974; Gentili and Huss-Danell, 2003). These studies suggest important interactions between P and N in regulating the N fixation behavior of legumes. However, the influence of the balance of N and P supply legume on plant physiology, root nodulation and symbiotic N fixation require further clarification. Increasing soil N availability may intensify P competition between host plant and nodule, because more soil available P may be assimilated by host plants to match its increasing growth and metabolism (Png et al., 2017). This competition could then inhibit symbiotic N fixation *via* direct reduction of P transfer into root nodules. However, previous studies indicated a strong P homeostasis in nodules (Sulieman and Tran, 2015), attributing preferential P allocation of legumes into nodules under P deficiency (Schulze and Drevon, 2005; Thuynsma et al., 2014). Relative P deficiency in soil can intensify P mobilization (increasing the availability of poorly soluble P forms) and uptake (Nuruzzaman et al., 2006; Betencourt et al., 2012; Png et al., 2017), by altering root development, root physiology and root-mycorrhizal interactions (Pang et al., 2018). These intensified P uptake mechanisms may increase competition between root nodules and the host legume or other symbionts for additional resources, such as photoassimilates (Vance et al., 2003; Ryan et al., 2012), that may also restrict root nodule development and symbiotic N fixation.

There are great inter-specific differences in response of symbiotic N fixation to changed soil nutrient availability (Nyfeler et al., 2011; Zheng et al., 2019). Such inter-specific differences make it complex to predict and evaluate N fixation capacity of leguminous plants in changing environments. However, the underlying mechanisms responsible for these inter-specific differences are poorly understood. It is well known that changes to soil N or P availability may induce changes in plant tissue chemistry (Lü et al., 2013; Li X. et al., 2016). However, plants have species-specific abilities to resist environmental change and maintain a relative stable inner element ratio (e.g. N:P ratio) termed stoichiometric homeostasis (Yu et al., 2011). The magnitude of stoichiometric homeostasis of N:P ratio can determine the variation in plant species N and P concentrations (Gusewell, 2004) and thus can be used to predict the plant growth and nutrient use strategies under changed soil N or P availability and subsequent N:P balance (Elser et al., 2010). Plant species with a greater stoichiometric homeostasis of N:P ratio potentially exhibit greater plant P deficiency under increasing N availability (Elser et al., 2010; Yu et al., 2010). As a response to N enrichment, these species can either acquire soil P more efficiently, for example, by exudation of P-mobilizing compounds (Pang et al., 2018), intensification of arbuscular mycorrhizal (AM)-plant interactions (Betencourt et al., 2012), releasing more P-acquiring enzymes (Png et al., 2017), or they can delay uptake of tissue N (Elser et al., 2010). The stoichiometric homeostasis index has been suggested as a reliable indicator to assess the responses of plants to changed soil nutrients status, in terms of plant nutrient uptake, allocation and resorption (Elser et al., 2010; Yu et al., 2011; Deng et al., 2016), as well as ecosystem structure and function (Yu et al., 2010; Yu et al., 2015). Symbiotic N fixation contributes to tissue N concentration of legume plants and is potentially regulated by the soil N:P balance. However, it remains unknown whether the stoichiometric homeostasis of plant N:P ratios can control those species-specific responses of symbiotic N fixation to altered N and P availability through regulation of plant N and P uptake. If found to be a valid model of plant nutrient interactions, its demonstration can improve our understanding and prediction of N cycling pattern at species and community level and assist in the practical management of legume crops to maximize agricultural N fixation benefits under changing soil nutrient status.

Accordingly, an outdoor pot experiment testing eight herbaceous legume species was established. This experiment examined the influence of soil N and P supply interactions on the symbiotic N fixation of eight legume species, inter-specific differences in symbiotic N fixation response to N enrichment, and tested whether those differences could be explained by the stoichiometric homeostasis in N:P ratios. We hypothesized that N enrichment decreases symbiotic N fixation of legumes by reducing P concentration in root nodules, and that legume species with a greater stoichiometric homeostasis of N:P ratio have greater reduction in specific symbiotic N fixation in response to increased N availability.

## 2 Materials and methods

### 2.1 Study site

A pot experiment was conducted in the field at the Songnen Grassland Research Station in Jilin province of northeastern China (E123˚31´, N44˚33´). This site has a semi-arid climate. From 2000 to 2016, mean annual temperature was 5.5°C, and annual precipitation was 404 mm, with 81% occurring between May and September. The soil is a meadow chernozem soil with a high pH (>8.5). *Leymus chinensis* (Trin.) Tzvel., a perennial C_3_ rhizomatous grass dominates the vegetation in this site.

### 2.2 Pot experiment design

Eight species of herbaceous legumes that co-occur in Songnen grasslands, consisting of’ four native species (*Medicago ruthenica* (L.) Sojak; *Lespedeza daurica* (Laxm.) Schindler; *Melilotus officinalis* (L.) Desr; *Glycine soja* Sieb. et Zucc), and four cultivated species (*Medicago sativa* L. cv. Aohan; *Medicago falcata* L. cv. Hulunbeier; *Medicago varia* Martin. cv. Caoyuan No.3; *Lotus corniculatus* L.), were grown separately in bottom-sealed plastic pots (0.3 m depth, 0.25 m diameter) in the field from June 2017 to September 2017. *L. chinensis* was also grown under the same conditions as a reference plant. Legume seeds were collected from a leguminous seed production field, while *L. chinensis* seeds were collected from a natural meadow at the Songnen Grassland Research Station in 2016. Cultivated soil was collected at the depth of 0 to 30cm from a natural *L. chinensis* meadow, then visible plant residues were removed and the soil was passed through a 2 mm sieve and evenly mixed. In total, eighteen kg soil (pH=8.74 ± 0.04; organic matter=17.3 ± 0.3 mg g^-1^; total N=1.26 ± 0.03 mg g^-1^; total P= 0.31 ± 0.02 mg g^-1^) was added into each pot, to achieve a bulk density of approximately 1.25 g cm^-3^, similar to that of the field site. In late May 2017, each species was sown into three replicate pots, with twelve pre-germinated seed sown, that were later thinned to four seedlings per pot. The seedlings in each pot were inoculated using crushed nodules specifically collected from the matching species in a seed production field site. Rather than inoculating plants with a single or a few known rhizobial strains, this inoculation approach increases ecological realism by allowing plant access to a broader range of naturally occurring rhizobia, and reduces the nodulation bias caused by artificially selected rhizobia, which has been used in several previous studies (Wolf et al., 2017; Li et al., 2021).

The experiment was a completely randomized block design with three replicates. In each block, four treatment combinations of two levels of P addition (0 and 3.5 g P m^-2^ as superphosphate) and two levels of N addition (0 and 14 g N m^-2^ as ammonium nitrate) were arranged across each plant species. The N addition rate was assumed to be sufficient to overcome plant N limitation in this grassland region (Zhan et al., 2017). P addition was defined according to the N addition rate and the mean N:P ratio in natural soil (0 to 30 cm depth). In this experiment, plants were grown for 84 d on average. For each pot, the total N and P fertilizer allocations were divided into three equal parts and applied at three times (on 0d, 30d and 60d since planting) dissolved in water. When rainfall occurred, the pots were protected from rainfall by setting up plastic shed during the whole experiment. Each pot was given 1000 ml water at an interval of 3 to 4 days. In each replicated block, pot placement was re-randomized weekly.

### 2.3 Measurement and sampling

Soil and plant samples for each legume species were harvested once the plants showed the first sign of flowering activity. Before collecting plant samples, leaf photosynthetic rate was determined on 2-3 mature and intact leaves per plant for each pot, using a using a CIRAS-2 potable photosynthetic measuring system (PP-Systems, Hitchin, UK). The intact soil core with plants was carefully separated from the pot. Legume plants with rhizosphere soil adhering were then removed from the core as it was gently broken up. The rhizosphere was defined as the volume of soil adhering up to approximately 2 mm from the root surface, which is suggested as the major P depletion zones of some legume plants (Nuruzzaman et al., 2006). Using a paintbrush, rhizosphere soil adhering to roots was gently brushed off. Then, the four plants were carefully separated from each other and the roots in each pot were lightly washed in the laboratory. Plant tissues were separated according to shoot, root and nodules for each plant and root nodule number was counted. Plant materials were subsequently dried at 60°C for 72h and weighed. From each pot, all tissues including shoot, root and root nodules from one randomized legume individual were combined and finely ground for analysis of tissue ^15^N abundance. Remaining shoots, roots, and nodules from each pot were combined according to tissue type, finely ground and analyzed for N and P concentrations, while the shoot and root samples were analyzed for carbohydrate concentration. The bulk soil was defined as remaining soil after rhizosphere soil sampling, which was also sampled after mixing uniformly in each pot.

### 2.4 Plant and soil analysis

Including shoot, root and root nodule, the entire plant sample of individual legumes and *L. chinensis* were analyzed for ^15^N natural abundance using a MAT253 stable isotope mass spectrometer (ThermoFisher Scientific, Waltham, USA). Total nitrogen (TN) concentration was analyzed using the Kjeldahl method (Sparks et al., 1996), and the total phosphorus (TP) concentration was analyzed using persulfate oxidation followed by colorimetric analysis for shoot and root samples (Schade et al., 2003). Non-structural carbohydrate (NSC) concentration in plant tissue was determined using an improved colorimetric method (Buysse and Merckx, 1993). Each rhizosphere soil sample was randomly divided into two parts; one part was transferred to a plastic bottle with 100 ml CaCl_2_ solution (0.2 μmol. L^-1^), the suspension was then frozen at -20℃ for organic acid analysis using the Ion Chromatography method (Baziramakenga et al., 1995), the other parts were oven-dried at 50℃ for 4 days and ground to pass through a 0.2 mm sieve. Soil available P concentration was determined by NaHCO_3_ extraction according to the Olsen method (Olsen et al., 1954). Soil pH of 1 g of soil extracted in ultrapure water was measured using a PHS-25 pH meter with a combined glass electrode (Leizi, Shanghai, China). The remaining bulk and rhizosphere soil sample was frozen at -20°C. ${\text{NH}}_{4}^{\;+}$ and ${\text{NO}}_{3}^{\;-}$ were extracted with 2 M KCl from this frozen soil sample (Miller and Keeney, 1982), and their concentrations were determined by a Bran-Luebbe AA3 autoanalyser (Bran and Luebbe, Hamburg, Germany). All soil nutrients were calculated based on units of dry soil weight.

### 2.5 Data analysis

The percentage of N in legume tissue derived from symbiotic N fixation (%Ndfa) was estimated according to the following formula (Unkovich et al., 2008):


(1) $$\delta \text{Ndfa}=100\times \left(\frac{{\text{ δ}}^{15}{\text{N reference plant-δ}}^{15}\text{N legume}}{{\text{δ}}^{15}\text{N reference plant-B}}\right)$$

Where δ ^15^N is the atom percentage excess ^15^N relative to atmospheric N. The term ‘legume’ represents the entire legume individual including shoot, root and nodule tissues, the term ‘reference plant’ represents whole *L. chinensis* individual including shoot and root grown under the same fertilization treatment as the legume. Here, *L. chinensis* was used as the reference species because it coexists with all experimental legume species and this species could represent the mean of four different reference plants in this study region in a previous study (Li et al., 2015); the “B” value reflects the within-plant fractionation of ^14^N and ^15^N between shoots and nodulated roots, which is represented as the δ ^15^N of legume tissues (e.g. shoot, root) fully dependent upon N_2_ fixation. Here, it is equal to the value of ^15^N atmosphere (0‰) because the whole legume individual was used as analysis (Unkovich et al., 2008). Symbiotic N fixation was calculated based on %Ndfa, legume tissue biomass and tissue N concentration. Specific symbiotic N fixation was calculated as symbiotic N fixation by unit legume biomass.

The P mobilization effect in the rhizosphere was calculated as the ratio of available P concentration in rhizospheric soil to available P concentration in bulk soil (Zhan et al., 2017). Stoichiometric homeostasis of plant N:P ratio (H_N:P_) is the ability of a plant to maintain a given tissue N:P ratio despite variation in the relative N:P availability of its environment (Wolf et al., 2017). The stoichiometric homeostasis index of plant N:P ratio (H_N:P_) was calculated according to the following equations (Yu et al., 2010):


(2) $$y=cx^{1/H}$$

where *y* is the N:P ratio of plants (without root nodules), *x* is the N:P ratio in the soil, *c* is a constant, *H* is the stoichiometric homeostasis index of plant N:P ratio.

All data were assessed to verify model assumptions of normality and equality of variance. Three-way ANOVA analysis was applied to determine the main and interaction effects of legume species, N and P addition on all measured variables. Mean comparisons were conducted by one-way ANOVA, followed by Duncan’s test. Pearson’s correlation coefficients were used to evaluate relationships between rhizosphere P mobilization, rhizosphere chemistry, AM colonization, root nodule biomass, specific symbiotic N fixation and root biomass and root NSC concentration. Regression analysis was performed to assess the relationships between H_N:P_ of legume species and its change in rhizosphere chemistry, leaf photosynthetic rate, tissue biomass, tissue NSC concentration, and specific symbiotic N fixation under N addition with or without P addition. Significance for all statistical tests was defined as *P ≤* 0.05. All data were analyzed using SPSS17.0 software (Chicago, IL, USA).

## 3 Results

### 3.1 Soil N and P availability, rhizosphere P mobilization and rhizosphere chemistry

**Figure 1:**
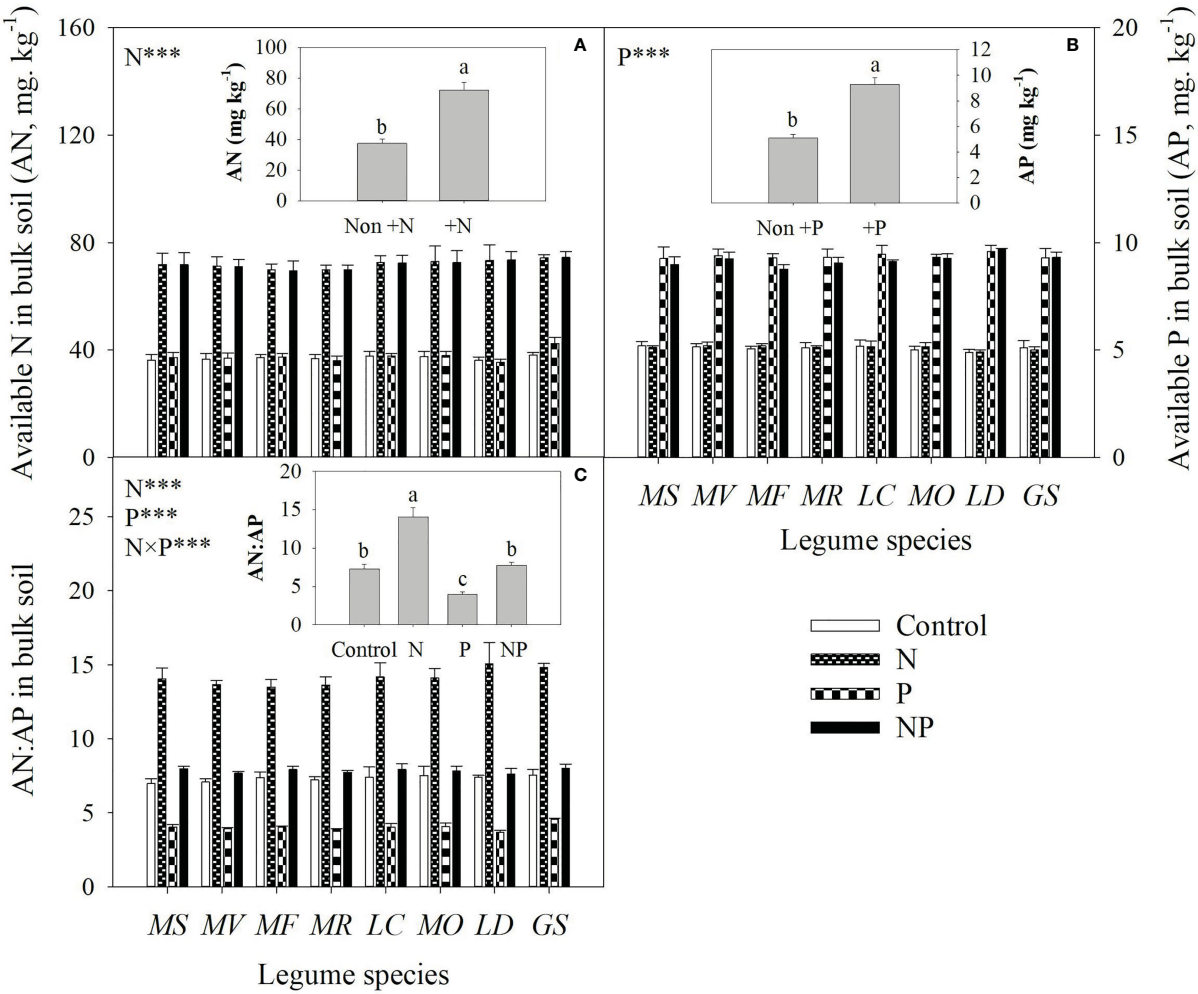
Available nitrogen (AN, **A**) and available phosphorus (AP, **B**) concentrations and AN : AP **(C)** in bulk soil under control (no fertilizer addition), N (N addition), P (P addition) and NP (addition of both N and P) for eight legume species. MS, *Medicago sativa*; MV, *Medicago varia*; MF, *Medicago falcata*; MR, *Medicago ruthenica*; LC, *Lotus corniculatus*; MO, *Melilotus officinalis*; LD, *Lespedeza daurica*; GS, *Glycine soja.* Letters (N, P, S, N×P, N×S, P×S, N×P×S) indicate significance overall from N addition, P addition, legume species (S) and their interaction effects according to general linear model P-values, the significance level are reported as ***(P<0.001). Inside the embedded figures, the bars represent the means of each independent factor and interacted treatment with significant effect.

**Figure 2:**
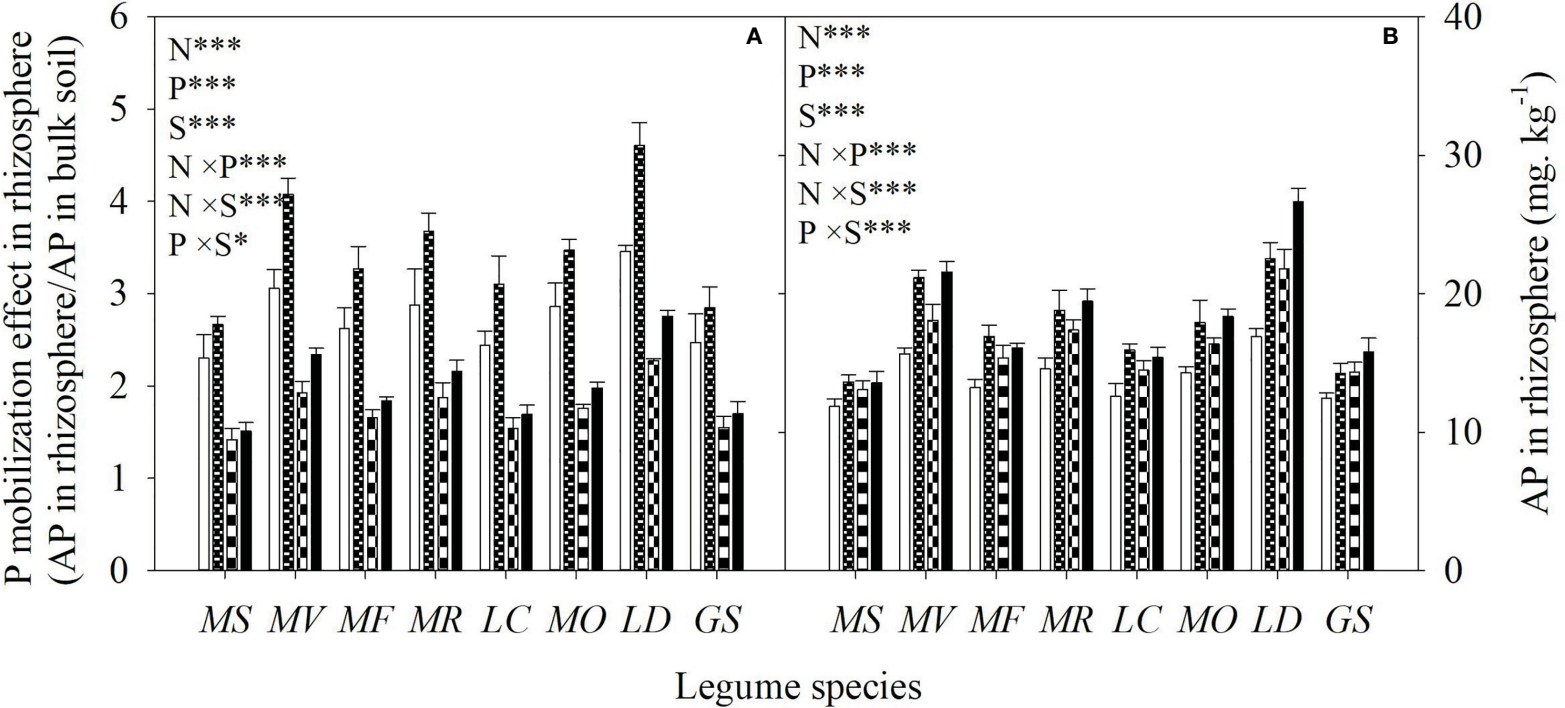
P mobilization effect in rhizosphere (ratio of AP in the rhizosphere to that in the bulk soil) **(A)** and available P (AP) concentration in rhizosphere soil **(B)** under control (no fertilizer addition), N (N addition), P (P addition) and NP (addition of both N and P) for eight legume species. MS, *Medicago sativa*; MV, *Medicago varia*; MF, *Medicago falcata*; MR, *Medicago ruthenica*; LC, *Lotus corniculatus*; MO, *Melilotus officinalis*; LD, *Lespedeza daurica*; GS, *Glycine soja.* Letters (N, P, S, N×P, N×S, P×S, N×P×S) indicate significance overall from N addition, P addition, legume species (S) and their interaction effects according to general linear model P-values, the significance level are reported as ***(P<0.001), **(P<0.01), *(P<0.05). Inside the embedded figures, the bars represent the means of each independent factor and interacted treatment with significant effect.

**Figure 3:**
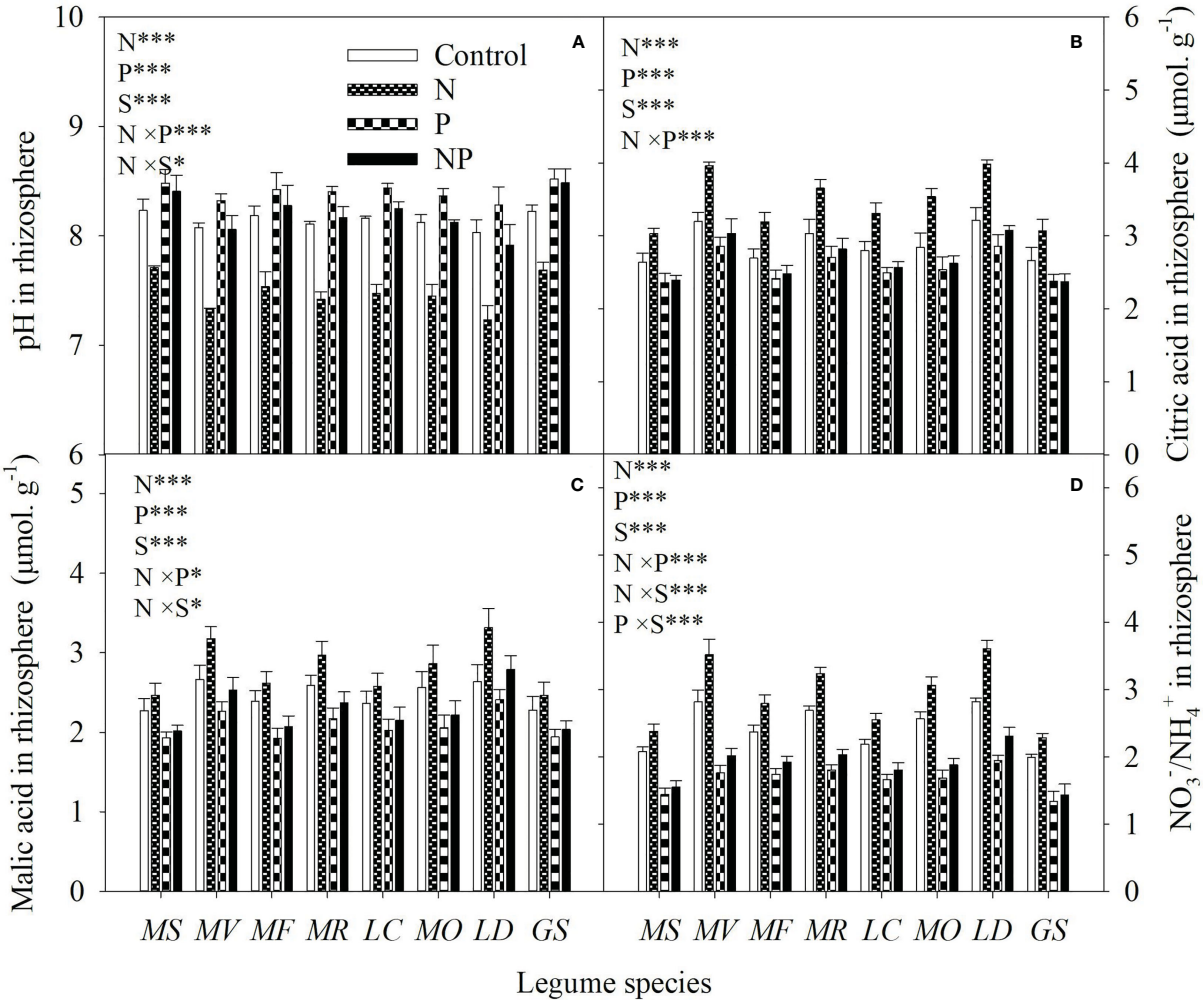
pH **(A)**, citric acid concentration **(B)**, malic acid concentration **(C)** and ${\text{NO}}_{3}^{\;-}$ / ${\text{NH}}_{4}^{\;+}$ ratio **(D)** in rhizosphere soil under control (no fertilizer addition), N (N addition), P (P addition) and NP (coupling addition of N and P) for eight legume species. MS, *Medicago sativa*; MV, *Medicago varia*; MF, *Medicago falcata*; MR, *Medicago ruthenica*; LC, *Lotus corniculatus*; MO, *Melilotus officinalis*; LD, *Lespedeza daurica*; GS, *Glycine soja.* Letters (N, P, S, N×P, N×S, P×S, N×P×S) indicate significance overall from N addition, P addition, legume species (S) and their interaction effects according to general linear model P-values, the significance level are reported as ***(P<0.001), **(P<0.01), *(P<0.05). Inside the embedded figures, the bars represent the means of each independent factor and interacted treatment with significant effect.

Averaged across all legume species, N and P addition increased available N and P concentrations of bulk soil by 93% and 82%, respectively. As a consequence, N addition increased the available N:P ratio, while P addition reduced the available N:P ratio in bulk soil (**Figure 1**). Compared with the control, N addition enhanced P mobilization in the rhizosphere by 15-33%, and thus increased the P availability in rhizosphere by 14-35% for eight legume species (**Figures 2A, B**). However, compared with P addition, the coupling addition of N and P enhanced P mobilization in the rhizosphere by 7-21%, and thus increased the P availability in rhizosphere by 4-22% for eight legume species (**Figures 2A, B**). N addition reduced soil pH, and increased citric acid and malic acid concentration and ${\text{NO}}_{3}^{\;-}$ / ${\text{NH}}_{4}^{\;+}$ ratio in the rhizosphere, and especially so without P addition (**Figure 3**).

### 3.2 Plant photosynthesis, biomass and NSC

**Figure 4:**
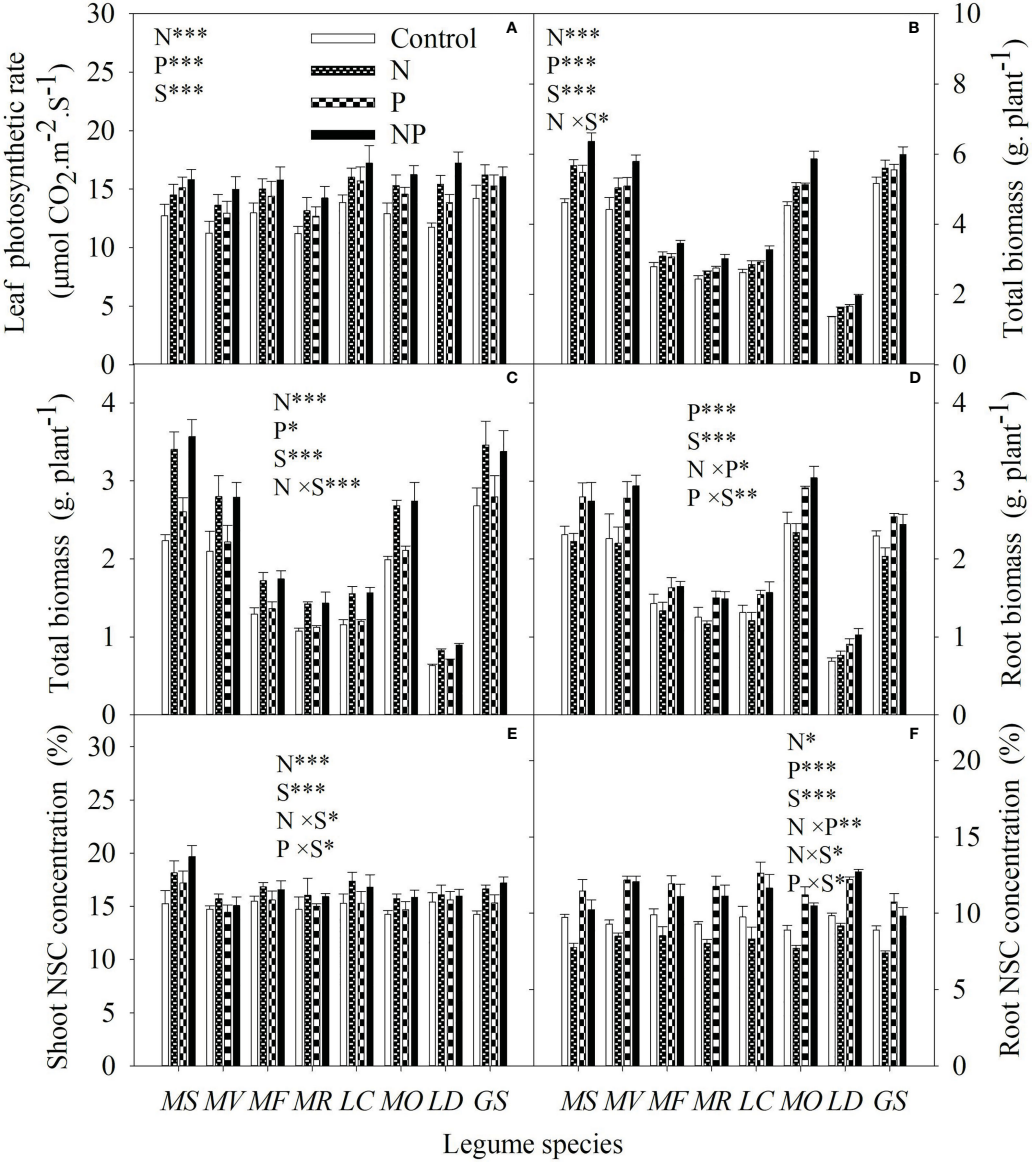
Leaf photosynthetic rate **(A)**, total biomass **(B)**, shoot biomass **(C)**, root biomass **(D)**, shoot **(E)** and root non-structural carbohydrate (NSC) concentration **(F)** under control (no fertilizer addition), N (N addition), P (P addition) and NP (addition of both N and P) for eight legume species. MS, *Medicago sativa*; MV, *Medicago varia*; MF, *Medicago falcata*; MR, *Medicago ruthenica*; LC, *Lotus corniculatus*; MO, *Melilotus officinalis*; LD, *Lespedeza daurica*; GS, *Glycine soja.* Letters (N, P, S, N×P, N×S, P×S, N×P×S) indicate significance overall from N addition, P addition, legume species (S) and their interaction effects according to general linear model P-values, the significance level are reported as ***(P<0.001), **(P<0.01), *(P<0.05). Inside the embedded figures, the bars represent the means of each independent factor and interacted treatment with significant effect.

Independent of P, N addition increased leaf photosynthetic rate, total biomass and shoot biomass (**Figures 4A–C**). N addition had no effect on root biomass except in concert with P addition (**Figure 4D**). N addition increased NSC concentration in shoots but decreased NSC concentration in roots without P addition (**Figures 4E, F**). For each legume species, there was significantly negative correlations between P mobilization effect, citric acid concentration, malic acid concentration, ${\text{NO}}_{3}^{\;-}$ / ${\text{NH}}_{4}^{\;+}$ ratio in the rhizosphere and root biomass or root NSC concentration, but significantly positive correlations between pH in rhizosphere and root biomass or root NSC concentration following nutrient additions (**Table 1**).

**Table 1:** Pearson correlation coefficients between root biomass (g.m^-2^), root non-structural carbohydrate (NSC) concentration (%) and rhizosphere P mobilization, pH in rhizosphere, citric acid concentration in rhizosphere (μmol. g^-1^), malic acid concentration in rhizosphere (μmol. g^-1^), ${\text{NO}}_{3}^{\;-}$ / ${\text{NH}}_{4}^{\;+}$ ratio in rhizosphere, arbuscular mycorrhizal (AM) colonization of root (%), root nodule biomass (mg. plant^-1^), specific symbiotic N fixation (mg N. g^-1^ plant) for each legume species.

	*Medicago sativa*		*Medicago varia*		*Medicago falcata*		*Medicago ruthenica*		*Lotus corniculatus*		*Melilotus officinalis*		*Lespedeza daurica*		*Glycine soja*	
	Root biomass	Root NSC	Root biomass	Root NSC	Root biomass	Root NSC	Root biomass	Root NSC	Root biomass	Root NSC	Root biomass	Root NSC	Root biomass	Root NSC	Root biomass	Root NSC
Rhizospheric P mobilization	-0.808^**^	-0.831^**^	-0.752^**^	-0.936^**^	-0.764^**^	-0.921^**^	-0.778^**^	-0.923^**^	-0.865^**^	-0.909^**^	-0.921^**^	-0.945^**^	-0.593^*^	-0.886^**^	-0.868^**^	-0.935^**^
pH in rhizosphere	0.617^*^	0.896^**^	0.546	0.747^**^	0.804^**^	0.919^**^	0.712^**^	0.885^**^	0.731^**^	0.824^**^	0.652^*^	0.853^**^	0.187	0.634^*^	0.941^**^	0.956^**^
Citric acid in rhizosphere	-0.822^**^	-0.931^**^	-0.603^*^	-0.811^**^	-0.609^*^	-0.789^**^	-0.777^**^	-0.801^**^	-0.679^*^	-0.768^**^	-0.720^**^	-0.839^**^	-0.623^*^	-0.771^**^	-0.954^**^	-0.929^**^
Malic acid in rhizosphere	-0.799^**^	-0.910^**^	-0.534	-0.820^**^	-0.770^**^	-0.785^**^	-0.797^**^	-0.797^**^	-0.623^*^	-0.673^*^	-0.841^**^	-0.820^**^	-0.099	-0.543	-0.919^**^	-0.905^**^
Root nodule biomass	0.497	0.815^**^	0.410	0.601^*^	0.447	0.603^*^	0.639^*^	0.790^**^	0.474	0.648^*^	0.520	0.825^**^	0.294	0.720^**^	0.794^**^	0.833^**^
Specific symbiotic N fixation	0.206	0.589^*^	0.880^**^	0.938^**^	0.479	0.637^*^	0.610^*^	0.932^**^	0.530	0.689^*^	0.839^**^	0.922^**^	0.852^**^	0.922^**^	0.695^*^	0.698^*^

*Correlation is significant at the 0.05 level (2-tailed),**Correlation is significant at the 0.01 level (2-tailed).

The bold values represent significant correlations.

### 3.3 N and P concentrations, and stoichiometry homeostasis of plant N:P ratio (H_N:P_)

**Figure 5:**
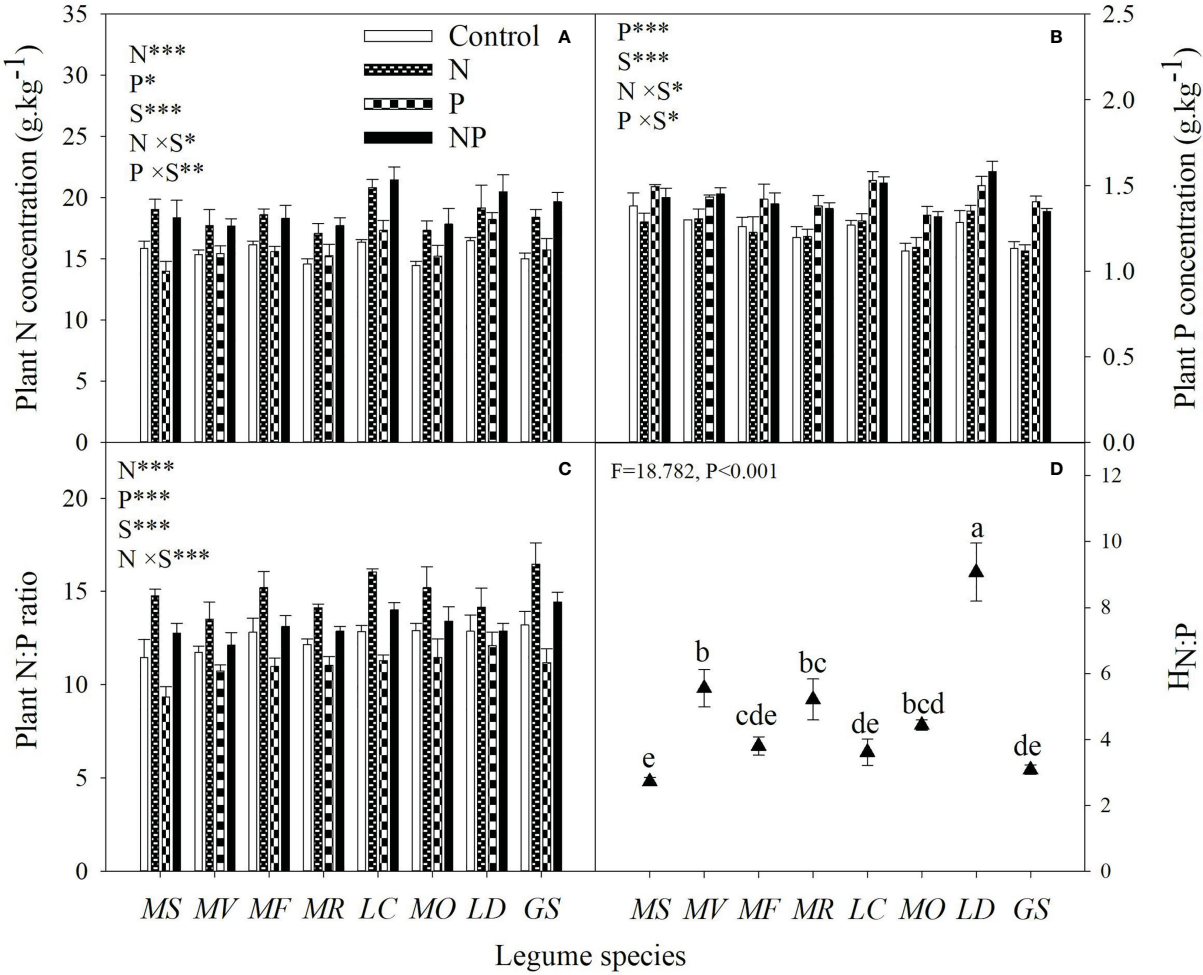
Plant N concentration **(A)**, plant P concentration **(B)**, plant N:P ratio **(C)** under control (no fertilizer addition), N (N addition), P (P addition) and NP (addition of both N and P), and stoichiometric homeostasis of plant N:P (H_N:P_, d) for eight legume species. MS, *Medicago sativa*; MV, *Medicago varia*; MF, *Medicago falcata*; MR, *Medicago ruthenica*; LC, *Lotus corniculatus*; MO, *Melilotus officinalis*; LD, *Lespedeza daurica*; GS, *Glycine soja.* Letters (N, P, S, N×P, N×S, P×S, N×P×S) indicate significance overall from N addition, P addition, legume species (S) and their interaction effects according to general linear model P-values, the significance level are reported as ***(P<0.001), **(P<0.01), *(P<0.05). Inside the embedded figures, the bars represent the means of each independent factor and interacted treatment with significant effect. Solid triangles without a common lowercase letter differed according to Duncan’s multiple comparison test (P<0.05).

N addition increased plant N concentration in all species, whereas P addition had negative, neutral or positive effects on plant N concentration (**Figure 5A**). Plant P concentration was increased by P addition, while its response varied from negative to positive with N addition in different legume species (**Figure 5B**). Averaged with and without P addition, N addition increased plant N:P ratio by 8% to 33% across legume species. Regardless of N addition and legume species, P addition decreased plant N:P ratio by an average of 12% (**Figure 5C**). The stoichiometric homeostasis of plant N:P ratio (H_N:P_) showed obvious inter-specific differences, ranging from 2.72 to 9.07 with the order of *L*. *daurica* > *M*. *varia* > *M*. ruthenica > *M*. *officinalis* > *M*. *falcata* > *L*. *corniculatus* > *G*. soja > *M*. *sativa* (**Figure 5D**).

### 3.4 Nodule number, nodule biomass and specific symbiotic N fixation

**Figure 6:**
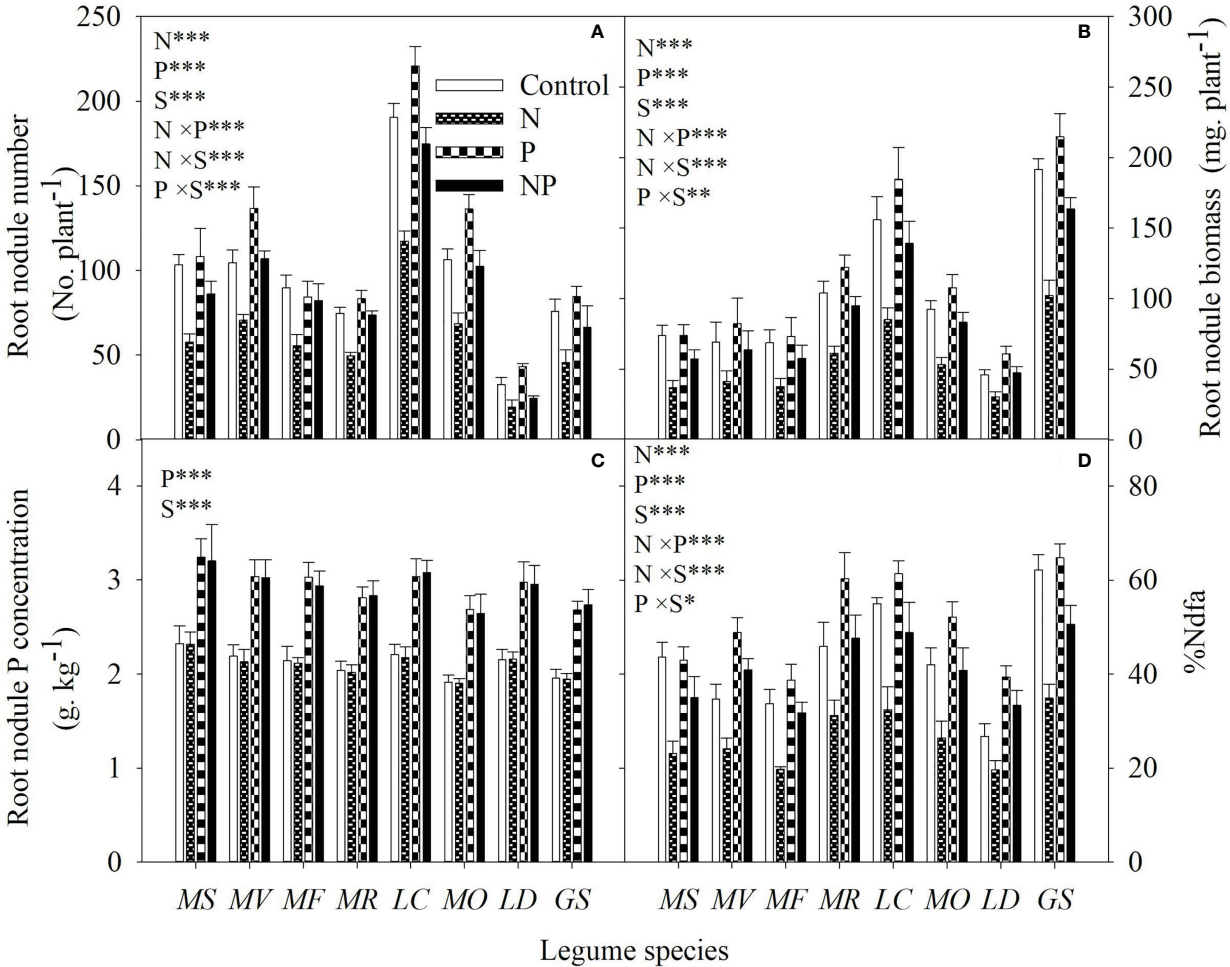
Root nodule number **(A)**, root nodule biomass **(B)**, root nodule P concentration **(C)** and specific symbiotic N fixation **(D)** under control (no fertilizer addition), N (N addition), P (P addition) and NP (addition of both N and P) for eight legume species. MS, *Medicago sativa*; MV, *Medicago varia*; MF, *Medicago falcata*; MR, *Medicago ruthenica*; LC, *Lotus corniculatus*; MO, *Melilotus officinalis*; LD, *Lespedeza daurica*; GS, *Glycine soja.* Letters (N, P, S, N×P, N×S, P×S, N×P×S) indicate significance overall from N addition, P addition, legume species (S) and their interaction effects according to general linear model P-values, the significance level are reported as ***(P<0.001), **(P<0.01), *(P<0.05). Inside the embedded figures, the bars represent the means of each independent factor and interacted treatment with significant effect.

Without P addition, N addition reduced the root nodule number, root nodule biomass and specific symbiotic N fixation by 32% to 44%, 34% to 50% and 14% to 36%, respectively; with P addition, N addition reduced root nodule number, root nodule biomass and %Ndfa by 2% to 43%, 22% to 25% and 2% to 14%, respectively, among the different legume species (**Figures 6A, B, D**). N addition and N-P interaction had no significant influence on root nodule P concentration (**Figure 6C**). Significantly positive correlations were also found between specific symbiotic N fixation and individual root biomass, or between specific symbiotic N fixation and root NSC concentration for all legume species (**Table 1**).

### 3.5 Correlation between H_N:P_ of legume species and its change in rhizosphere chemistry, biomass and NSC, and specific symbiotic N fixation

**Figure 7:**
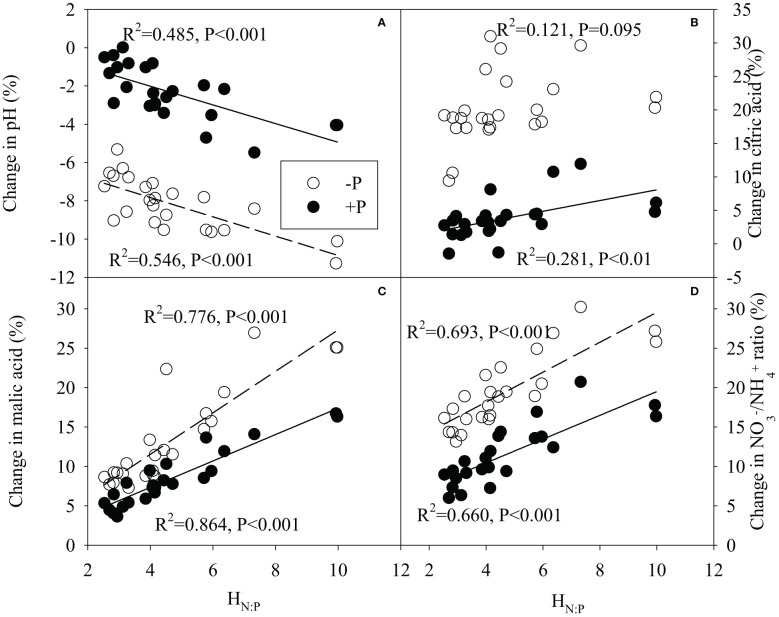
Correlation relationships between stoichiometric homeostasis of plant N:P (H_N:P_) of legume species and its change in pH **(A)**, citric acid concentration **(B)**, malic acid concentration **(C)** or ${\text{NO}}_{3}^{\;-}$ / ${\text{NH}}_{4}^{\;+}$ ratio **(D)** in rhizosphere under N addition with or without P addition.

**Figure 8:**
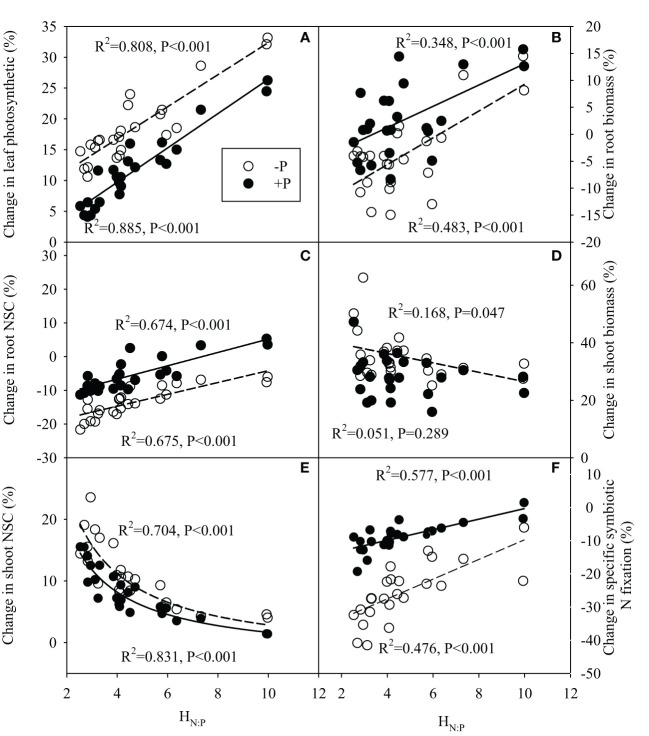
Correlation relationships between stoichiometric homeostasis of plant N:P (H_N:P_) of legume species and its change in leaf photosynthetic rate **(A)**, root biomass **(B)**, root non-structural carbohydrate (NSC) concentration **(C)**, shoot biomass **(D)**, shoot non-structural carbohydrate (NSC) concentration **(E)** and %Ndfa **(F)** under N addition with or without P addition.

Regression analysis generally showed significantly positive correlations between H_N:P_ of legume species and changes in malic acid concentration, ${\text{NO}}_{3}^{\;-}$ / ${\text{NH}}_{4}^{\;+}$ ratio in rhizosphere, and significantly negative correlations between H_N:P_ of legume species and its change in rhizosphere pH under N addition, regardless of P additions (**Figure 7**). Meanwhile, there were significantly positive correlations between H_N:P_ of legume and its changes in leaf photosynthetic rate, root biomass, root NSC concentration following N addition with or without P addition (**Figures 8A–D**). N-driven change in shoot biomass for legume species was negatively correlated with its H_N:P_ without P addition (**Figure 8D**). Regardless of P addition, the change in shoot NSC concentration for legume species showed the reciprocal relationship with its H_N:P_ under N addition (**Figure 8E**). With or without P addition, the H_N:P_ value of legume species were positively correlated with its change in specific symbiotic N fixation following N addition (**Figure 8F**).

## 4 Discussion

### 4.1 Legumes species responses to N and P additions, and their H_N:P_ values

An increase in either N or P availability can drive plants to adjust nutrient utilization and tissue chemistry for adaptation to nutrient imbalances (Reich et al., 2009; Li X. et al., 2016). In this study, N addition primarily increased plant N concentrations, with less influence on plant P concentrations, consequently causing a significant increase in plant N:P ratio across legume species. However, N addition greatly increased plant biomass, therefore the stable plant P concentration indicates that legume species enhanced P acquisition to balance the increased biomass P demand (Li X. et al., 2016; Png et al., 2017). Moreover, as observed in this study, the effect of P addition on plant N concentration, as well as the effect of N addition on plant P concentration, showed significant inter-specific differences, suggesting that species differences appear to influence the responses of plant tissue chemistry to the changes in soil N and P availability. Similarly, previous studies have suggested that the species-specific changes in tissue nutrients could occur across different plant function groups in response to varied N and P availability (Betencourt et al., 2012; Mayor et al., 2014; Lü et al., 2016; Li X. et al., 2016). The present study also demonstrated that the response of plant tissue chemistry is not phylogenetically conserved even within taxonomic family/functional group under changed environment (Wolf et al., 2017). According to our results, *Lespedeza daurica* showed a much more positive response in P uptake compared with other legume species when challenged with N enrichment. Eventually, because of species-specific responses in N and P uptakes, the changes of plant N:P ratio showed great inter-specific difference under N addition.

The stoichiometric homeostasis in plant N:P ratio reflect different abilities of legumes to maintain tissue N:P stability during unbalanced nutrient supply (Yu et al., 2010). Previously, only limited data were reported about the H_N:P_ of legumes (Wolf et al., 2017). In the present study, the H_N:P_ values of eight measured legume species, especially *Lespedeza daurica*, were evidently higher than those previously reported for other legumes (1.8-2.7; Wolf et al., 2017), suggesting plant N:P ratio for legume species could be more conserved at the intraspecific level than that previously assessed, and implying that P may have increasing importance in regulating legume growth under N enrichment. Meanwhile, our results indicated that the H_N:P_ values of legume species had greater inter-specific variability than that previously estimated (Wolf et al., 2017), implying that the ratio of N and P fertilizer application particularly needs to be adjusted according to species for optimizing legume production (Wolf et al., 2017).

### 4.2 The effects of N and P in regulating symbiotic N fixation

N addition inhibited root nodulation and symbiotic N fixation in legumes, supporting previous research (West et al., 2005; Nyfeler et al., 2011; Zheng et al., 2019). However, N addition had a less inhibitory effect on symbiotic N fixation when the experimental soil was supplemented with P fertilizer, suggesting the importance of plant P status in mediating the influence of N enrichment on symbiotic N fixation (Gates and Wilson, 1974; Gentili and Huss-Danell, 2003). In this study, N addition increased plant biomass and tissue N concentration, which enhanced the legume demands for P (White and Hammond, 2008; Png et al., 2017). Since external P supply did not increase following N addition without P supplementation, this N/P supply imbalance created a potential P limitation to legume growth. It was originally expected that P limitation reduced symbiotic N fixation through increasing the P competition between host legume and rhizobium, and thus reducing P transfer into root nodules. However, root nodule P was maintained at relatively stable concentration under N addition. This likely excludes the possibility that P limitation reduced P nutrition of the root nodule, and thus symbiotic N fixation in response to N additions. However, the stabilization of P levels in symbiotic tissues may be a common mechanism for allowing nodules to ameliorate the negative effects of P deficiency (Sulieman and Tran, 2015). To meet the needs of concurrent stabilization of P levels in host plants and symbiotic tissues under increased biomass by N addition, legumes likely increased P acquisition *via* adjustment in P uptake mechanisms (Pang et al., 2018).

Intensification of root-mycorrhizal interactions can be an important mechanism for legumes to enhance P acquisition (Betencourt et al., 2012; Ryan et al., 2012; Pang et al., 2018). However, in another study using the same cultivated soil, legume species and N addition treatment, it has been confirmed that these legume species did not improve P acquisition by intensifying root-mycorrhizal interaction (Li et al., 2021). Alternatively, increasing root phosphatase activity may also greatly help legumes to acquire more P under N enrichment (Pang et al., 2018). However, root phosphatase activity generally had positive and neutral feedback with symbiotic N fixation across legumes (Png et al., 2017; Li et al., 2021), implying increased root phosphatase activity will not reduce the symbiotic N fixation. In the present study, N addition clearly stimulated P mobilization in the rhizosphere by which legumes improve P acquisition under N addition. Rhizosphere acidification of plants as observed in the current study can be a key mechanism that increases P mobilization under N addition, especially in alkaline soils, such as those in this study (Hinsinger et al., 2003; Zhou et al., 2009). Several physiological responses of roots may have contributed to decline of rhizosphere pH, including increased exudates of P-mobilizing compounds such as organic acids, and increased efflux of H^+^, which can be driven by more uptake of ${\text{NH}}_{4}^{\;+}$ -N than ${\text{NO}}_{3}^{\;-}$ -N in rhizosphere (Hinsinger et al., 2003; Blossfeld and Gansert, 2007; Bilyera et al., 2022), or increased root respiration (Nuruzzaman et al., 2006; Li et al., 2007). Current results showed significant negative correlations between rhizosphere P mobilization, rhizosphere acidification and root biomass and root carbohydrate concentration, because efflux of P-mobilizing compounds (e.g., organic acids) and rhizosphere acidification likely accounted for a vast consumption of carbohydrates in the root (Lynch et al., 2005; Ryan et al., 2012). As our results imply, root biomass and their carbohydrate content are important in the regulation of root nodule development and activity, providing carbon skeletons and energy to nodules (Sulieman et al., 2013; Divito and Sadras, 2014). Therefore, the N/P imbalance in soil by N addition likely has inhibited symbiotic N fixation *via* increased P mobilization in rhizosphere.

When P was supplemented, N additions induced lesser increases in rhizosphere P mobilization, and subsequently less decline in root biomass and root carbohydrate concentration. Correspondingly, specific symbiotic N fixation was less inhibited by N addition when the experimental soil received P fertilizer. Similarly, the increase in N fixation when P was supplied could also be due to the host no longer having to rely on P mobilization mechanisms to scavenge P, which potentially allowed more carbon skeletons to be available for N assimilation. These results provide further evidence to suggest a legume trade-off between rhizosphere P mobilization and symbiotic N fixation under changed N-P balance in soil, and explain a mechanism by which P availability regulates symbiotic N fixation inhibited by N enrichment.

### 4.3 Stoichiometric homeostasis of N:P ratio drives species-specific symbiotic N fixation inhibition

As observed in present research, species-specific inhibition of symbiotic N fixation in response to increased soil N availability is widely reported (Nyfeler et al., 2011; Zheng et al., 2019). However, the underlying mechanisms responsible for these inter-specific differences have not been adequately explored. Ecological stoichiometry potentially provides a tool to address this research gap, as it services as a basis framework to control the feedback between biology and environment (Elser et al., 2010; Yu et al., 2015; Zheng et al., 2020). Previously, many studies have revealed the control of substrate stoichiometry to biological N fixation in forest or agriculture ecosystems, and found that the greater decline in substrate C:N and C:(N:P) ratios generally drove the more decline in biological N fixation rate under N addition (Reed et al., 2011; Zheng et al., 2020). However, these studies have not explained why legumes showed species-specific inhibition of symbiotic N fixation with the same change in substrate nutrient availability.

In this study, as N addition increased tissue N concentration and biomass of legumes, legume species with a less change (greater stoichiometric homeostasis) of plant N:P ratio had a greater P demand that will provide a greater stimulation of P mobilization in the rhizosphere under N addition (Elser et al., 2010; Yu et al., 2010). This is supported by our finding that H_N:P_ of legume was positively correlated with rhizosphere acidification and changes of organic acid concentration and ${\text{NO}}_{3}^{\;-}$ / ${\text{NH}}_{4}^{\;+}$ ratio in the rhizosphere following N addition. Accordingly, legume species with a greater stoichiometric homeostasis of N:P ratio is expected to have a greater symbiotic N fixation inhibition under N addition because increased P mobilization may cause a greater decline in root biomass and root carbohydrate concentration (Lynch et al., 2005; Ryan et al., 2012). However, in contrast to our previous hypothesis, the current results indicate that legume species that have a greater stoichiometric homeostasis of N:P ratio, instead have less reduction of symbiotic N fixation with N addition. That is because the legume species with a greater stoichiometric homeostasis of N:P ratio appear to have less decline, and even a weak increase in root biomass and root carbohydrate concentrations under N addition. It seems paradoxical that legume species with a greater increase in rhizosphere P mobilization have less decline in root biomass and root carbohydrate concentration. Further analysis explained the potential reasons: first, high H_N:P_ species increased more sharply in plant photosynthetic rate under N addition, with greater increase in carbohydrate production compared to species with a lower H_N:P_. In addition, a greater increase of plant photosynthesis and carbohydrate production potentially means greater promotion of biomass and carbohydrate accumulation in legume tissues. However, our results showed that legume species with greater H_N:P_ had less increase in shoot biomass and shoot carbohydrate concentration under N addition, implying that high H_N:P_ legumes likely increase more photosynthate allocation into roots under N addition, compensating for the loss of root carbohydrate by rhizosphere P mobilization. Finally, as a key rhizosphere acidification mechanism, the greater increase of rhizosphere ${\text{NO}}_{3}^{\;-}$ / ${\text{NH}}_{4}^{\;+}$ ratio was found in legume species with greater H_N:P_ values under N addition. There is no evidence to indicate that changes in ${\text{NO}}_{3}^{\;-}$ / ${\text{NH}}_{4}^{\;+}$ uptake of legume are physiologically relevant to loss of root biomass and carbohydrate. Therefore, high H_N:P_ legumes may enhance the other rhizosphere acidification mechanism, such as increased relative uptake of ${\text{NH}}_{4}^{\;+}$ -N to ${\text{NO}}_{3}^{\;-}$ -N in the rhizosphere, potentially inhibiting the cost of root biomass and carbohydrate under N addition. To sum up, the legume species with greater H_N:P_ have more capacity to maintain root biomass and root carbohydrate concentration *via* regulation in carbon and nitrogen assimilation, consequently reducing the symbiotic N fixation inhibition under N addition.

## 5 Conclusions

This research demonstrated that N addition can reduce the symbiotic N fixation of legumes by increasing the relative P limitation for growth, which stimulates rhizosphere P mobilization at the expense of root biomass and carbohydrate concentrations that regulate root nodule development and nutrition. P addition alleviates the relative P limitation induced by N addition, reducing symbiotic N fixation suppression under N enrichment. Legume species that had more stoichiometric homeostasis in plant N:P ratio have less reduction in symbiotic N fixation under N addition.

In conclusion, this research identified the trade-off between rhizosphere P mobilization and symbiotic N fixation as a mechanism to account for changes in symbiotic N fixation by legumes under changing N availability, and demonstrated species-specific stoichiometric control of their symbiotic N fixation response. Future research to improve legume performance should consider stoichiometric homeostasis of plant N:P ratio to provide a predictive tool for understanding how legumes respond to changes in soil nutrient status, to attain a greater symbiotic N fixation contribution to benefit soil fertility and health.

## Data availability statement

The raw data supporting the conclusions of this article will be made available by the authors, without undue reservation.

## Author contributions

QL was responsible for conception, field sampling and manuscript draft writing. JP, MD, and JW was responsible for manuscript revision. YH was responsible for data analysis. HS, YL, and QZ was responsible for sampling and analysis. All authors contributed to the article and approved the submitted version.
